# 1521. Non-faecium Non-faecalis enterococcal bloodstream infections in patients with cancer

**DOI:** 10.1093/ofid/ofac492.083

**Published:** 2022-12-15

**Authors:** Dierdre B Axell-House, Stephanie L Egge, Cecilia Tran, Samuel A Shelburne, Cesar A Arias

**Affiliations:** Houston Methodist Research Institute, Houston, Texas; Houston Methodist Hospital/Houston Methodist Research Institute, Houston, Texas; Houston Methodist Hospital, Houston, TX; MD Anderson, Houston, Texas; Houston Methodist Hospital and Weill Cornell Medical College, Houston, TX

## Abstract

**Background:**

Non-*E. faecium* non-*E. faecalis* (NFF) enterococci are a heterogeneous group of organisms consisting of enterococci with intrinsic low-level vancomycin resistance as well as several other species known to infect humans. Patients with cancer are at increased risk for bloodstream infections (BSIs) with NFF enterococci, but their optimal treatment and outcomes have not been extensively described.

**Methods:**

We conducted a retrospective review of patients (pts) with blood cultures positive for all NFF by searching the microbiology database at a major cancer center in Houston, TX from 2016 to 2021. Patients were included if they were ≥ 18 years old, had ≥ 1 blood culture with NFF enterococci, and had a repeat blood culture within 7 days (d) of the index culture, to permit outcome analysis. Outcomes were a) in-hospital mortality, b) microbiological failure (blood culture clearance >4 d after index culture), and c) recurrence of bacteremia (new positive blood culture < 14 d after eradication).

**Results:**

Sixty-two unique pts had NFF enterococcal BSI. Patients with hematological malignancy made up 53.2% of the cohort (78% had leukemia). The majority (83%) of solid malignancies were pancreatic, biliary, or intestinal in origin. In the 30 d preceding NFF enterococcal BSI, 79% of pts had exposure to antibiotics. The NFF species isolated were *E. gallinarum* (50%), *E. casseliflavus* (30.6%), *E. avium* (11.3%), *E. raffinosus* (3.2%), *E. hirae* (3.2%), and *E. durans* (1.6%). Only 23.3% of NFF enterococci were susceptible to vancomycin, and 53.8% were susceptible to linezolid. Most (62.9%) pts received combination therapy; the most frequent was daptomycin plus a beta-lactam (33.9%). Bacteremia recurred in 8.1% of pts, 27.4% had in-hospital mortality, and 4.8% had microbiological failure.

**Conclusion:**

This retrospective review provides preliminary insight into the treatments, and outcomes of cancer pts with NFF enterococcal BSIs. The NFF enterococci isolates in this study had a higher rate of linezolid non-susceptibility than previously reported. Molecular investigation of these NFF enterococcal isolates is needed to elucidate the mechanisms and transmission of antibiotic resistance. Comparison of BSIs caused by NFF enterococci to *E. faecium*/*E. faecalis* will provide insight to physicians caring for these pts.

**Disclosures:**

**Cesar A. Arias, MD, PhD**, Entasis: Grant/Research Support|MeMed Diagnostics Ltd: Grant/Research Support|Merck: Grant/Research Support.

